# Clinical and laboratory characteristics in juvenile-onset systemic lupus erythematosus across age groups

**DOI:** 10.1177/0961203320909156

**Published:** 2020-03-31

**Authors:** J S Massias, E M D Smith, E Al-Abadi, K Armon, K Bailey, C Ciurtin, J Davidson, J Gardner-Medwin, K Haslam, D P Hawley, A Leahy, V Leone, F McErlane, D Mewar, G Modgil, R Moots, C Pilkington, A V Ramanan, S Rangaraj, P Riley, A Sridhar, N Wilkinson, M W Beresford, C M Hedrich

**Affiliations:** 1 School of Medicine, 4591 University of Liverpool, UK; 2 Department of Women's & Children's Health, Institute of Translational Medicine, 4591 University of Liverpool, UK; 3 Department of Paediatric Rheumatology, Alder Hey Children's NHS Foundation Trust Hospital, UK; 4 Department of Rheumatology, Birmingham Children’s Hospital, Birmingham, UK; 5 Department of Paediatric Rheumatology, Cambridge University Hospitals, Cambridge, UK.; 6 Department of Paediatric Rheumatology, 6397 Oxford University Hospitals NHS Foundation Trust, Oxford, UK; 7 Department of Rheumatology, University College London Hospitals NHS Foundation Trust, London, UK; 8 Department of Paediatric Rheumatology, 59841 Royal Hospital for Sick Children, Edinburgh, UK; 9 Department of Child Heath, 3526 University of Glasgow, Glasgow, UK; 10 Department of Paediatrics, 111991 Bradford Royal Infirmary, Bradford, UK; 11 Department of Paediatric Rheumatology, Sheffield Children’s Hospital, Sheffield, UK; 12 Department of Paediatric Rheumatology, 105634 Southampton General Hospital, Southampton, UK; 13 Department of Paediatric Rheumatology, Leeds Children Hospital, Leeds, UK; 14 Paediatric Rheumatology, Great North Children’s Hospital, 105563 Royal Victoria Infirmary, Institute of Cellular Medicine, Newcastle University, Newcastle upon Tyne, UK; 15 Department of Rheumatology, 159020 Royal Liverpool University Hospital, Liverpool, UK; 16 Department of Paediatrics, 156775 Musgrove Park Hospital, Taunton, UK; 17 Department of Rheumatology, 89542 University Hospital Aintree, Liverpool, UK; 18 Department of Paediatric Rheumatology, Great Ormond Street Hospital, London, UK; 19 University Hospitals Bristol NHS Foundation Trust & Bristol Medical School, University of Bristol, Bristol, UK; 20 Department of Paediatric Rheumatology, Nottingham University Hospitals Nottingham, UK; 21 Department of Paediatric Rheumatology, Royal Manchester Children’s Hospital, Manchester, UK; 22 Department of Paediatrics, 156756 Leicester Royal Infirmary, Leicester, UK; 23 Guy's & St Thomas's NHS Foundation Trust, Evelina Children's Hospital, London, UK

**Keywords:** Age group, childhood, juvenile-onset SLE, phenotype, SLE

## Abstract

**Background:**

Systemic lupus erythematous (SLE) is a systemic autoimmune/inflammatory condition. Approximately 15–20% of patients develop symptoms before their 18th birthday and are diagnosed with juvenile-onset SLE (JSLE). Gender distribution, clinical presentation, disease courses and outcomes vary significantly between JSLE patients and individuals with adult-onset SLE. This study aimed to identify age-specific clinical and/or serological patterns in JSLE patients enrolled to the UK JSLE Cohort Study.

**Methods:**

Patient records were accessed and grouped based on age at disease-onset: pre-pubertal (≤7 years), peri-pubertal (8–13 years) and adolescent (14–18 years). The presence of American College of Rheumatology (ACR) classification criteria, laboratory results, disease activity [British Isles Lupus Assessment Group (BILAG) and Systemic Lupus Erythematosus Disease Activity Index 2000 (SLEDAI-2 K) scores] and damage [Systemic Lupus International Collaborating Clinics (SLICC) damage index] were evaluated at diagnosis and last follow up.

**Results:**

A total of 418 JSLE patients were included in this study: 43 (10.3%) with pre-pubertal disease onset; 240 (57.4%) with peri-pubertal onset and 135 (32.3%) were diagnosed during adolescence. At diagnosis, adolescent JSLE patients presented with a higher number of ACR criteria when compared with pre-pubertal and peri-pubertal patients [pBILAG2004 scores: 9(4–20] vs. 7(3–13] vs. 7(3–14], respectively, *p* = 0.015] with increased activity in the following BILAG domains: mucocutaneous (*p* = 0.025), musculoskeletal (*p* = 0.029), renal (*p* = 0.027) and cardiorespiratory (*p* = 0.001). Furthermore, adolescent JSLE patients were more frequently ANA-positive (*p* = 0.034) and exhibited higher anti-dsDNA titres (*p* = 0.001). Pre-pubertal individuals less frequently presented with leukopenia (*p* = 0.002), thrombocytopenia (*p* = 0.004) or low complement (*p* = 0.002) when compared with other age groups. No differences were identified in disease activity (pBILAG2004 score), damage (SLICC damage index) and the number of ACR criteria fulfilled at last follow up.

**Conclusions:**

Disease presentations and laboratory findings vary significantly between age groups within a national cohort of JSLE patients. Patients diagnosed during adolescence exhibit greater disease activity and “classic” autoantibody, immune cell and complement patterns when compared with younger patients. This supports the hypothesis that pathomechanisms may vary between patient age groups.

## Introduction

Systemic lupus erythematosus (SLE) is a systemic autoimmune/inflammatory condition that can affect any organ system and result in significant damage and organ failure.^1,2^ Clinical characteristics, underlying pathomechanisms, disease progression and outcomes vary between individuals, age groups and races. Approximately 15–20% of SLE patients develop the disease before their 18th birthday and are therefore diagnosed with juvenile-onset SLE (JSLE).^1,2^ Juvenile-onset disease is associated with more severe organ involvement (including renal and CNS disease), increased disease activity, presence of greater damage at the time of diagnosis, and higher steroid burden, contributing to the increased morbidity and mortality when compared with adult-onset SLE^3–5^. Even within the JSLE population, very early disease onset (before the 5th birthday) may be associated with atypical presentations (including fewer autoantibodies), more severe disease course and poor prognosis.^1,6–8^ However, assumptions on variable disease presentation and progression within different JSLE age subgroups are generally based on case reports, case series or relatively small cohorts,^7,8^ and currently lack scientific evidence from longitudinal national or international studies.

Preliminary datasets suggest that clinical differences may reflect variable pathomechanisms and that patients with JSLE may have increased genetic burden when compared with individuals with adult-onset disease, contributing to early disease onset and more severe presentations.^1,9^ Very early disease onset, atypical disease presentation and severe manifestations may be the result of (very rare) disease-causing mutations in single genes or the combination of multiple genomic variants that individually increase an individual’s risk for the development of SLE.^1,9–11^ To date, evidence still remains weak and it is largely unclear whether distinct clinical and laboratory differences exist between age groups within the paediatric population.^1,12,13^

This study aimed to assess if there are differential clinical and laboratory characteristics in patients presenting with JSLE at different ages, subdividing patients into three groups: pre-pubertal (≤7 years), peri-pubertal (8–13 years) or adolescence (14–18 years). To achieve this, prospectively collected data from a national cohort of JSLE patients (the UK JSLE Cohort Study) was interrogated.

## Methods

### Patients

Participants of the UK JSLE Cohort Study,^14^ followed between 2006 and 2018, aged ≤16 years at the time of diagnosis and with at least four American College of Rheumatology (ACR) classification criteria for SLE,^15^ were included in this study. Participants were excluded from the study if they did not have a diagnosis date recorded, as this precluded them from being categorized on the basis on their age at disease-onset [pre-pubertal (≤7 years), peri-pubertal (8–13 years) or adolescent (14–18 years)]. Patient/family-reported ethnicity information was collected using UK National Census categories.^12^ Data of patients of mixed race were grouped with those of the associated ethnic minority group (e.g. Asian if mixed Asian and Caucasian race). Of note, distribution among ethnicities did not vary between age groups (see *Results*, supplemental Tables S1 and S2).

### Data collected

The following clinical and laboratory data were collected: (a) total ACR score with its individual domains;^15^ (b) antinuclear antibody (ANA) positivity and titre; (c) Systemic Lupus International Collaborating Clinics standardised damage index (SLICC-SDI) score;^16^ (d) Systemic Lupus Erythematosus Disease Activity Index 2000 (SLEDAI-2 K) score;^17^ (e) paediatric British Isles Lupus Assessment Grade 2004 numerical scores (pBILAG2004) with individual organ/system domains (alphabetical score A–E)^18^; (f) key laboratory findings, including haemoglobin levels, white cell count and differentiation, platelets, erythrocyte sedimentation rate (ESR), complement levels (C3, C4) and anti-double-stranded DNA (dsDNA) titres.

The SLICC-SDI tool records permanent damage that occurs as a result of SLE activity, and is composed of 41 different components.^16^ The SLEDAI- score is a widely used measure of lupus disease activity, providing numerical scores based on a count of laboratory and clinical symptoms.^17^ The pBILAG2004 score is a composite disease activity measure focusing on nine organ/system domains (constitutional, mucocutaneous, neurological, musculoskeletal, cardiovascular/respiratory, renal, gastrointestinal, ophthalmic and haematological). Each organ domain is graded A–E and defined as follows; pBILAG2004 grade A/B: severe and moderate disease respectively, grade C patients: mild/improving renal disease, grade D: inactive disease but previous system involvement, grade E: system has never been involved.^19,20^ For each organ/system domain, an alphabetical (A–E grade) is determined, equating to a numerical value for each organ/system domain. These can be combined to give the total numerical pBILAG2004 score.^18^ Within these analyses, presence of pBILAG2004 domains A and B was taken to signify active organ/system involvement, in keeping with previous studies.^18^ All data items (a–e listed above) were collected at the time of initial diagnosis. At the patients’ last follow-up visit, data from items a and d were collected. Furthermore, data from item e were collected from patients as their cumulative maximum disease activity level (for each individual organ/system domain) throughout the disease course.

### Statistical analysis

Laboratory findings, total number of ACR criteria, SLICC, SLEDAI-2 K and pBILAG2004 scores were compared between groups using Kruskal-Wallis tests. Median values and interquartile ranges (IQRs) are displayed within tables. Categorical pBILAG2004 domain data is presented as a percentage of patients with active organ/system involvement for each age group along with 95% confidence intervals. Individual domains of the pBILAG2004 score were compared between groups using Chi-square and Fisher’s exact tests. Analyses were completed using SPSS software, version 25 (IBM SPSS).

Power analysis revealed that the three patient groups should all have approximately 700 patients each to reach sufficient statistical power. Limited by the rarity of JSLE and resulting number of patients included in the national UK JSLE cohort study since 2006, these numbers are extremely difficult to obtain in national, or even international, cohorts. Thus, p values of statistical tests should be interpreted with caution, based upon the limited statistical power of this study.

### Ethics

Written patient assent/consent and/or, where appropriate, parental consent was obtained for inclusion of patients within the UK JSLE Cohort Study. The UK JSLE Cohort Study has full ethical approval from the National Research Ethics Service North West, Liverpool East (REC reference 06/Q1502/77). This research was carried out in accordance with the decleration of Helsinki.

## Results

### Demographics

A total of 418 eligible patients enrolled in the UK JSLE Cohort Study were included in this study; five JSLE patients were excluded due to unknown age at diagnosis. The mean age at diagnosis was 12.1 years (range: 0.17–17.91), with 43/418 (10.3%) participants presenting in the pre-pubertal period, 240/418 (57.4%) were peri-pubertal, and 135/418 (32.3%) were in the adolescent age group. The overall female:male ratio was 5.4:1 and increased with age (pre-pubertal = 3.3:1; peri-pubertal = 5.24:1; adolescent = 7.25:1). No statistically significant differences were demonstrated between groups in relation to ethnicity (*p* > 0.05) (Supplemental Tables S1 and S2).

### Clinical features

At diagnosis, adolescent JSLE patients exhibited higher median ACR scores when compared with younger JSLE patients [pre-pubertal: median 4(IQR 4–5) vs. peri-pubertal: 4(4–5) vs. adolescent: 5(4–6), p = 0.004]. Similarly, pBILAG2004 disease activity scores were higher in newly diagnosed adolescent JSLE patients [pBILAG2004: 9(4–20)] when compared with younger JSLE patients [pre-pubertal: 7(3–13); peri-pubertal: 7(3–14), *p* = 0.015] (Table 1). First SLEDAI-2 K scores were also higher in the adolescent population [pre-pubertal: 8(4–14); peri-pubertal 8(4–14); adolescent 12(6–18), *p* = 0.001] (Table 1). Furthermore, adolescents with a new diagnosis of JSLE exhibited more activity in the following pBILAG domains when compared with new peri-pubertal and pre-pubertal JSLE patients: mucocutaneous (*p* = 0.025), musculoskeletal (*p* = 0.029), cardiorespiratory (*p* = 0.001) and renal (*p* = 0.027) (Table 1).

**Table 1:** Clinical features of JSLE subgroups at diagnosis

Item	Pre-pubertal disease-onset (*n* = 43)	Peri-pubertal disease-onset (*n* = 240)	Adolescence (*n* = 135)	*p* value
Female:male ratio	3.3:1	5.24:1	7.25:1	0.347
Total ACR score	4 [4–5]	4 [4–5]	5 [4–6]	0.004
SLICC-SDI	0 [0]	0 [0]	0 [0]	0.410
SLEDAI-2 K	8 [4–14]	8 [4–14]	12 [6–18]	0.001
Total numerical pBILAG2004 score	7 [3–13]	7 [3–14]	9 [4–20]	0.015
Active organ/system involvement at diagnosis (pBILAG2004 defined)				
• Constitutional	13 (30.2%) [16.5%, 43.9%]	67 (27.9%) [22.2%, 33.6%]	51 (37.8%) [29.6%, 46.0%]	0.140
• Mucocutaneous	19 (44.2%) [29.5%, 59.0%]	78 (32.5%) (26.6%, 38.4%]	62 (45.9%) [37.5%, 54.3%]	0.025
• Neuropsychiatric	6 (40.0%) [25.4%, 54.6%]	20 (8.3%) [4.8%, 11.8%]	14 (10.4%) [5.3%, 15.3%]	0.477
• Musculoskeletal	7 (16.3%) [5.3%, 27.3%]	66 (27.5%) [21.9%, 33.1%]	49 (36.3%) [28.2%, 44.4%]	0.029
• Cardiorespiratory	4 (9.3%) [0.6%, 18%]	18 (7.5%) [4.2%, 10.8%]	27 (20%) [13.3%, 26.7%]	0.001
• Gastrointestinal	2 (4.7%) [0%, 11%]	15 (6.3%) [3.2%, 9.4%]	4 ((3.0%) [0.1%, 5.9%]	0.442
• Ophthalmic	1 (2.3%) [0%, 6.8%]	2 (0.8%) [0%, 1.9%]	1 (0.7%) [0%, 2.1%]	0.548
• Renal	9 (20.9%) [8.7%, 33.1%]	73 (30.4%) [24.6%, 36.2%]	55 (40.7%) [32.4%, 49.0%]	0.027
• Haematological	11 (25.6%) [12.6%, 38.6%]	58 (24.1%) [18.7%, 29.5%]	37 (27.4%) [19.9%, 34.9%]	0.786

Total ACR, SLICC-SDI, SLEDAI-2 K, pBILAG2004 scores, and key laboratorial findings are reported as median values and interquartile ranges [in square brackets]. For individual pBILAG2004 organs/systems involved, the total number of patients with active involvement (defined as pBILAG2004 domain score of A or B within a given organ domain/system) is provided along with the percentage (in curved brackets) and 95% confidence intervals for the percentage [in square brackets].

ACR, American College of Rheumatology; BILAG, British Isles Lupus Assessment Group; JLSE, juvenile onset systemic lupus erythematous; SLICC-SDI, Systemic Lupus International Collaborating Clinics-standardised damage index; SLEDAI-2 K, Systemic Lupus Erythematosus Disease Activity Index 2000.

At the time of last follow up, differences were not apparent between age groups in terms of total ACR scores [median of 5(IQR 4–7) in all groups], disease activity shown through SLEDAI-2 K [pre-pubertal: 8(5–8); peri-pubertal: 8(6–8); adolescent 7(5–8), p = 0.689] or antinuclear antibodies (ANA) positivity. Over the disease course active organ/system involvement (as defined by the pBILAG2004 score) also did not differ significantly between age groups (Table 2). There was little variance in SLICC-SDI defined damage at diagnosis (*p* = 0.410) or last follow-up (*p* = 0.284) between age groups (Tables 1 and 2).

**Table 2:** Clinical features of the different age groups over time

Items on last follow up	Pre-pubertal disease-onset (*n* = 43)	Peri-pubertal disease-onset (*n* = 240)	Adolescence (*n* = 135)	*p* value
Length of follow up in years	6 [3–9]	4 [2–6]	2 [1–4]	<0.001
Total ACR score	5 [4–7]	5 [4–7]	5 [4–7]	0.686
SLICC-SDI score	0 [0–1]	0 [0–1]	0 [0–1]	0.284
SLEDAI-2 K	8 [5–8]	8 [6–8]	7 [5–8]	0.689
ANA positivity	42 (97.7%) [93.2%, 102.2%]	230 (95.8%) [93.3%, 98.3%]	132 (97.8%) [95.3%, 100.0%]	0.559
pBILAG2004 defined organ/system domain involvement throughout the disease course				
Constitutional	18 (41.9%) [27.2%, 56.6%]	98 (40.8%) [34.1%, 46.5%]	56 (41.5%) [33.2%, 49.8%]	0.988
Mucocutaneous	33 (76.7%) [64.1%, 89.3%]	157 (65.4%) [59.4%, 71.4%]	90 (66.7%) [58.7%, 74.7%]	0.346
Neuropsychiatric	11 (25.6%) [12.6%, 38.6%]	57 (27.9%) [22.2%, 33.6%]	28 (20.7%) [13.9%, 27.5%]	0.731
Musculoskeletal	18 (41.9%) [16.5%, 43.9%]	121 (50.4%) [44.1%, 56.7%]	73 (54.1%) [45.7%, 62.5%]	0.374
Cardiorespiratory	13 (30.2%) [16.5%, 43.9%]	46 (19.2%) [14.2%, 24.2%]	35 (25.9%) [18.5%, 33.3%]	0.141
Gastrointestinal	8 (18.6%) [7.0%, 30.2%]	28 (11.7%) [7.6%, 15.8%]	10 (7.4%) [3.0%, 11.8%]	0.107
Ophthalmic	3 (7.0%) [0%, 14.6%]	12 (5.0%) [2.2%, 7.8%]	4 (3.0%) [0.1%, 5.9%]	0.467
Renal	28 (65.1%) [50.9%, 79.3%]	153 (63.8%) [57.7%, 69.9%]	94 (69.6%) [61.8%, 77.4%]	0.513
Haematological	26 (60.5%) [45.9%, 75.1%]	114 (47.5%) [41.2%, 53.8%]	55 (40.7%) [32.4%, 49.0%]	0.072

Total ACR, SLICC-SDI, SLEDAI-2 K, pBILAG2004 scores are reported as median values and interquartile ranges. For individual pBILAG2004 domains, the total number of patients with activity involvement (defined as a pBILAG2004 domain score of A or B in a given organ domain/system) are provided along with percentage (in curved brackets), and 95% confidence intervals for the percentage [square brackets]. SLICC-SDI and ACR scores are provided from the last follow-up visit.

ACR, American College of Rheumatology; ANA, antinuclear antibodies; BILAG, British Isles Lupus Assessment Group; SLICC-SDI, Systemic Lupus International Collaborating Clinics-standardised damage index; SLEDAI-2 K, Systemic Lupus Erythematosus Disease Activity Index 2000.

### Laboratory features

Laboratory findings varied between JSLE patients from different age groups at diagnosis (Table 3). White blood cell and platelet counts reduced with growing age across the JSLE cohort; with pre-pubertal patients exhibiting median white cell counts of 6.7 × 10^9^/L [4.69–9.53] vs. 6.09 × 10^9^/L [4.16–8.67] in peri-pubertal vs. 4.69 × 10^9^/L [3.7–6.54] in the adolescent age group (*p* = 0.002). Median platelet counts were within the normal range, but followed a similar pattern to the white cell count, with 293 × 10^9^/L [212–426] in the pre-pubertal group vs. 271 × 10^9^/L [191–388] in the peri-pubertal vs. 242 × 10^9^/L [168–298] in the adolescent group (*p* = 0.004). Median levels of haemoglobin (*p* = 0.404) and ESR (*p* = 0.2) did not differ between age groups (Table 3).

**Table 3:** Laboratory features of JSLE subgroups at diagnosis

Key laboratory findings	Pre-pubertal disease-onset (*n* = 43)	Peri-pubertal disease-onset (*n* = 240)	Adolescence (*n* = 135)	*p* value
Haemoglobin level (g/dL)	11 [9–11.9]	11.3 [9.9–12.6]	11.08 [9.7–12.53]	0.404
White cell count (×10^9^/L)	6.7 [4.69–9.53]	6.09 [4.16–8.67]	4.69 [3.7–6.54]	0.002
Platelets (×10^9^/L)	293 [212–426]	271 [191–338]	242 [168–298]	0.004
ESR (mm/hr)	18 [11–72]	36 [12–76]	42.5 [19–86.75]	0.200
C3 median (g/L)	0.95 [0.73–1.11]	0.81 [0.50–1.22]	0.69 [0.28–0.98]	0.002
C4 median (g/L)	0.13 [0.08–0.28]	0.11 [0.06–0.19]	0.08 [0.04–0.14]	0.002
ANA positive	37 (86.0%) [80.7%, 91.3%]	223 (92.9%) [89.7%, 96.2%]	131 (97.0%) [94.1%, 99.9%]	0.034
ANA titre median	1:640 [1:320–1:960]	1:640 [1:320–1:1280]	1:640 [1:320–1:2560]	0.565
dsDNA levels (IU/L)	15 [0.25–89]	67 [19–200]	111 [15–300]	0.001

Haemoglobin, white cell count, platelets, ESR, C3, C4, ANA titre and dsDNA titre are reported as median values and interquartile ranges [in square brackets].

ANA, antinuclear antibodies; CSR, central serous retinopathy; JLSE, juvenile onset systemic lupus erythematous.

Serum complement is a measure of disease activity in SLE as it indicates activation and consumption of complement components,^21^ Median complement levels differed significantly between age groups, with higher complement levels in younger patients [C3: 0.95 g/L (0.73–1.11) in pre-pubertal patients vs. 0.81 g/L (0.50–1.22) in peri-pubertal vs. 0.69 g/L (0.28–0.98) in adolescent patients (p = 0.002); C4: 0.13 g/L (0.08–0.28) in pre-pubertal patients vs. 0.11 g/L (0.06–0.19) peri-pubertal patients vs. 0.08 g/L (0.04–0.14) in adolescent patients (*p* = 0.002)] (Table 3).

In the UK JSLE cohort, patients with disease-onset during adolescence were more frequently ANA-positive 131/135 (97.0%) at diagnosis, when compared with the other age groups; 37/43 (86.0%) with pre-pubertal onset, and 223/240 (92.9%) in peri-pubertal onset (*p* = 0.034). Anti-dsDNA antibody titres were higher in older patients than younger patients; pre-pubertal onset 15 IU/L (0.25–89) vs. 67 IU/L (19–200) in peri-pubertal group vs. 111 IU/L (15–300) in adolescents (*p* = 0.001) (Table 3).

## Discussion

While clinical and laboratory differences between JSLE and adult-onset SLE have been acknowledged,^8^ only few and short reports discuss differences within the paediatric age group.^12,13^ The 418 JSLE patients included in this study allow for more reliable assessment of clinical and laboratory features between the paediatric age groups. When compared with younger children, adolescents exhibit an increased number of ACR criteria, and show typical autoantibody patterns (ANA and anti-dsDNA positivity), haematological involvement (leukopenia, thrombocytopenia) and immunological characteristics (hypocomplementaemia) reflecting “classical” SLE. Of note, adolescents also present with higher disease activity at diagnosis when compared with younger children (total numerical BILAG score, *p* = 0.015; and SLEDAI-2 K scores, *p* = 0.001). At diagnosis, differences were also seen in the organ domains involved across age groups, including increased mucocutaneous, musculoskeletal, cardiorespiratory and renal system involvement in adolescents when compared with other age groups. Notably, previous studies did not consider pre-pubertal (≤7) JSLE patients as a distinct age group.^3,6–8^

One of the most interesting differences between JSLE patients within the three age groups relates to laboratory findings. Patients diagnosed in early childhood (≤7 years) had lower rates of ANA positivity, with 14% of the pre-pubertal JSLE patients being ANA negative vs. 3% of the adolescent JSLE group (*p* = 0.034). Pre-pubertal children also displayed lower median anti-dsDNA titres than the other age groups (*p* = 0.001). These laboratory differences may reflect differences in pathophysiology at varying ages, and a potentially more “innate” disease phenotype in at least a subset of early-onset JSLE patients.^1^

Of note, previous studies failed to identify serological differences between paediatric and adult SLE populations, which may be due to them not discriminating between age groups within the JSLE population^3,^ ^8,^ ^22^. This potential explanation is supported by the observation that differences in immunological patterns (ANA positivity) disappeared by the time of last follow up prior to transition into adult care (*p* = 0.559). Most patients who were initially autoantibody negative in the pre-pubertal (11.7%) and peri-pubertal age groups (2.9%), eventually developed ANA positivity (pre-pubertal group: 14% at diagnosis vs. 2% at last follow up) between the time of initial diagnosis and last follow-up. It has previously been discussed that early-onset JSLE patients, who may have a higher genetic risk when compared with older SLE patients or have a more monogenic disease phenotype, can develop autoantibodies over time as a result of tissue damage, and subsequent presentation of physiologically nuclear components to the immune system.^1,23^

This study also found increased frequencies of ANA positivity to coincide with an increased prevalence of likely autoantibody-mediated symptoms, e.g. renal, musculoskeletal and haematological anomalies (thrombocytopenia, lymphopenia and low complement levels, all *p* < 0.05). Autoantibodies (particularly anti-dsDNA antibodies) indeed contribute to renal disease and immune complex deposition, which may also partially cause the pathologically reduced complement levels observed with increasing age.^2,21,24^ Also, increased musculoskeletal involvement in adult-onset SLE vs. JSLE patients has been previously demonstrated.^3,8,13^ Tavangar-Rad *et al.* studied 120 Iranian children with JSLE, and compared age groups in a similar way to the current study (<7, 7–14, and >14 years) and reported more joint involvement with increasing age.^13^ While it remains unclear why this is, musculoskeletal involvement is another example of a clinical feature that may be auto-antibody driven, thus becoming more prevalent with advancing age at presentation.

Findings from this study also suggest that disease activity within the paediatric age group may (at diagnosis) be more severe in individuals diagnosed in adolescence, while disease severity increases over time in children diagnosed at a younger age. This is indicated by comparable disease activity and damage scores at last follow up. Based on variable clinical patterns over time that coincide with increased disease activity, autoantibodies, immune complex deposition, and complement activation may likely be involved in this process.^2,21,24^ Differences between the present study and previous reports suggesting increased disease severity in very early-onset SLE when compared with “older” children with JSLE, may be due to the character of previous reports.^12^ Small case series and individual case reports tend to over-report particularly severe, interesting and/or complicated presentations and disease courses.

The absence of ANA antibodies in 14% of pre-pubertal JSLE patients is interesting when considering the classification criteria for SLE. Recently proposed “new” ACR/EULAR criteria for SLE include ANA titres of ≥1:80 as entry criterion^25^. While application of these criteria would affect only a relatively small number of peri-pubertal or adolescent JSLE patients, 14% of patients with early disease-onset could potentially remain without a diagnosis, as classification criteria are frequently (incorrectly) used by colleagues (not necessarily specialized in paediatric rheumatology) to diagnose SLE and refer to tertiary care. One may argue that very early disease-onset in the absence of autoantibodies can indicate genetic conditions (“monogenic SLE-like disease”, such as complement deficiencies, primary type I interferonopathies) and that it is beneficial for patients to not be classified as “classical” SLE. However, this may result in diagnostic delays and young patients not being seen by paediatric rheumatologists.^26^

Although this study involves one of the largest national JSLE cohorts available, it is still limited by JSLE being a rare disease with low patient numbers. A power analysis performed prior to this study suggested that around 700 patients were required per group for the analysis to be statistically reliable. Since the UK JSLE cohort study is the largest JSLE cohort across Europe and one of the largest in the world, this limitation can unfortunately currently not be addressed. International collaboration is therefore warranted in the future. The variable duration of follow up from initial evaluation to last visit between the three age groups may also be seen as a potential limitation. This was caused mainly by the time of transition to adult care.

## Conclusion

This is the largest study to date comparing clinical and laboratory features of JSLE patients diagnosed during the pre- (≤7), peri-pubertal (8–13) and adolescent (14–18) periods. Distinct clinical and laboratory differences between age groups support the hypothesis that variable pathomechanisms may contribute to differences in clinical presentations, treatment responses and disease outcomes, not only between adult and paediatric patients but also within the cohort of JSLE patients. Based on the presence of autoantibodies and higher prevalence of antibody-mediated features (including thrombocytopenia, lymphopenia, hypocomplementaemia), adaptive immune mechanisms may play an increasing role with growing age. Disease activity at diagnosis is higher in individuals diagnosed in adolescence when compared with younger patients. However, disease severity increases over time in children diagnosed at a younger age underscoring the importance of tightly monitored and sufficient treatment in a specialised centre. Though the largest study of its kind, it is still limited by patient numbers, due to the rarity of JSLE. Thus, international collaborations are warranted to address age-specific differences in JSLE in more detail.

## Supplemental Material

LUP909156 Supplemental Material - Supplemental material for Clinical and laboratory characteristics in juvenile-onset systemic lupus erythematosus across age groups Click here for additional data file. Supplemental material, LUP909156 Supplemental Material for Clinical and laboratory characteristics in juvenile-onset systemic lupus erythematosus across age groups by J S Massias, E M D Smith, E Al-Abadi, K Armon, K Bailey, C Ciurtin, J Davidson, J Gardner-Medwin, K Haslam, D P Hawley, A Leahy, V Leone, F McErlane, D Mewar, G Modgil, R Moots, C Pilkington, A V Ramanan, S Rangaraj, P Riley, A Sridhar, N Wilkinson, M W Beresford and C M Hedrich in Lupus
