# Supplementary material for: Clinical and laboratory characteristics in juvenile-onset systemic lupus erythematosus across age groups
Source: Lupus. 2020 Mar 31;29(5):474–81. doi: 10.1177/0961203320909156 (PMC7528537; doi:10.1177/0961203320909156)
Supplement: LUP909156 Supplemental Material - Supplemental material for Clinical and laboratory characteristics in juvenile-onset systemic lupus erythematosus across age groups [file LUP909156_Supplemental_Material.pdf]

Supplement table 1 – Ethnicity of participants

Ethnicity	Pre-pubertal disease-onset (n=43)	Peri-pubertal disease-onset (n=240)	Adolescence (n=135)	P value
British or Irish	19/43 (44.2%) [29.3%, 59.0%]	104/240 (43.3%) [37.0%, 49.6%]	77/135 (57.0%) [48.7%, 65.4%]	0.100
Asian	13/43 (30.2%) [16.5%, 44.0%]	73/240 (30.4%) [24.6% ,36.2%]	41/135 (30.4%) [22.6%, 38.1%]	
African/Caribbean	8/43 (18.6%) [7.0%, 30.2%]	45/240 (18.8%) [13.8%, 23.7%]	14/135 (10.4%) [5.2%, 15.5%]	
Other Caucasian origin	1/43 (2.3%) [-2.2%, 6.8%]	7/240 (2.9%) [0.8%, 5.0%]	2/135 (1.5%) [-0.6% ,3.5%]	
Any other mixed/black background	0/43 (0%) [0%, 0%]	7/240 (2.9%) [0.8%, 5.0%]	0/135 (0%) [0%, 0%]	
Ethnicity not stated	2/43 (4.7%) [-1.6%, 10.9%]	4/240 (1.67%) [0.0%, 3.3%]	1/135 (0.7%) [-0.7%, 2.2%]	

Ethnicities were compared between age groups using the Chi Square test of independence. For each ethnic group, the total number of patients is provided along with the percentage (in curved brackets), and 95% confidence intervals for the percentage [in square brackets].

Supplement table 2: Distribution of ethnicities across age groups.

Age groups	*White Caucasian*	*Asian*	*Afro-Caribbean*	*No of individuals*	*P value*
≤7	20 (48.8%)	13 (31.7%)	8 (19.5%)	41	
8 to 13	111 (47.0%)	74 (31.4%)	51 (21.6%)	236	
≥14	79 (59.0%)	41 (30.6%)	14 (10.4%)	134	0.072
No of individuals	210	127	74	411	

Differences in ethnical compositions among sub-cohorts was tested using Chi square tests.
